# Design and conduct of a full diet-controlled, parallel, 2-week residential trial for diabetes prevention without weight loss in Asian Chinese and European Caucasian adults with prediabetes: the New Zealand SYNERGY study

**DOI:** 10.3389/fnut.2025.1590579

**Published:** 2025-06-19

**Authors:** Ivana R. Sequeira-Bisson, Karl Fraser, Kok Hong Leiu, Jack Penhaligan, Aidan Joblin-Mills, Lindsay D. Plank, Rinki Murphy, Michael W. Taylor, Olivier Gasser, Denise M. Conroy, Yannan Jiang, Louise W. W. Lu, Sally D. Poppitt, Jennifer L. Miles-Chan

**Affiliations:** ^1^Human Nutrition Unit, School of Biological Sciences, Faculty of Science, University of Auckland, Auckland, New Zealand; ^2^High Value Nutrition National Science Challenge, Auckland, New Zealand; ^3^Riddet Institute, Massey University, Palmerston North, New Zealand; ^4^Food Chemistry & Structure Team, AgResearch, Palmerston North, New Zealand; ^5^Department of Surgery, University of Auckland, Auckland, New Zealand; ^6^Department of Medicine, University of Auckland, Auckland, New Zealand; ^7^Auckland Diabetes Centre, Health New Zealand, Te Whatu Ora, Auckland, New Zealand; ^8^Maurice Wilkins Centre for Molecular Biodiscovery, University of Auckland, Auckland, New Zealand; ^9^School of Biological Sciences, University of Auckland, Auckland, New Zealand; ^10^Malaghan Institute of Medical Research, Wellington, New Zealand; ^11^New Zealand Institute for Plant and Food Research Ltd, Auckland, New Zealand; ^12^Department of Statistics, Faculty of Science, University of Auckland, Auckland, New Zealand

**Keywords:** residential study, full-diet control, ethnicity, prediabetes, ectopic fat, faecal microbiome, plasma metabolomics, indirect calorimetry

## Abstract

**Background:**

The causal underpinning of increased metabolic risk and previously observed dichotomous plasma metabolome in Asian Chinese vs. European Caucasian remains undetermined and may be hypothesised as attributed to ethnicity (genetic background), pathology (dysglycaemia) and/or lifestyle (habitual diet). We aimed to investigate the underlying cause(s) and the effect of dietary intervention on biomarkers of type 2 diabetes (T2D) in cohorts with prediabetes. The diets are a generic current Best Practice Healthy Diet (‘BPHD’), and a New Zealand-specific healthy diet (‘SYNERGY’) based on the Mediterranean Diet. We hypothesise, firstly, that 14-days of matched BPHD in Asian Chinese vs. European Caucasian cohorts (ethnicity; within-diet comparison) will attenuate the previously observed dichotomy in plasma metabolome. Secondly, that both diets will improve risk markers over 14 days vs. baseline, with significant improvement with SYNERGY compared to BPHD in Asian Chinese cohorts (diet; within-ethnicity comparison).

**Methods:**

We conducted a 2-week, fully diet-controlled, residential trial in 20 Asian Chinese (*n* = 10 per diet group) and 10 European Caucasian (BPHD only) adults with prediabetes. Participants were phenotyped (dual-energy X-ray absorptiometry, magnetic resonance imaging/spectroscopy) prior to the intervention. On Day 1 (D1) and D15 assessments included anthropometry, collection of urine, faecal (microbiome analysis) and fasted blood samples, conduct of 2-h oral glucose tolerance test (established clinical, metabolome, immune markers) and indirect calorimetry (resting metabolic rate, postprandial glucose-induced thermogenesis). Additional fasted urine and blood samples were collected on D2, D7 (mid-way) and D14, with a focus group/interview on the evening of D7. Meals and snacks were calculated based on individual energy requirements for body weight maintenance, dietary compliance was supervised, and body weight monitored daily.

**Discussion:**

This study aims to identify ethnic-specific dietary responses in a fully-controlled residential setting; to determine cause/s of the dichotomous plasma metabolome between the two ethnic groups; also to validate these biomarkers as sensitive to dietary intervention using a ‘whole of diet’ approach. Specifically, to determine the efficacy of BPHD and SYNERGY for T2D risk amelioration in the absence of body weight loss. Findings will inform design of larger ‘free-living’ community interventions and explore the feasibility of use of these diets within the community.

**Clinical trial registration (SPIRIT 2a):**

The study was prospectively registered on 22 March 2021 with the Australian New Zealand Clinical Trials Registry ACTRN12621000318886.

## 1 Introduction (SPIRIT 6a & b)

The exponential growth in global obesity rates (1), and concomitant prevalent ‘*tsunami*’ of type 2 diabetes (T2D) (2), poses a considerable public health challenge, with efforts focussed on developing strategies (3) to mitigate the associated economic burden. An added complexity requiring critical consideration is the unequivocal evidence from recent studies (4–6), including that from our group (7–10), which highlight the importance of ethnic-specific variability in susceptibility to developing prediabetes and T2D, even at a younger age (11) and in the absence of obesity (12, 13).

Whilst gain in body weight and adiposity (14) and unhealthy dietary and lifestyle factors (15) disrupt energy homeostasis and lipo-regulation, it is the adverse promotion of adipose tissue deposition in metabolically ‘risky’ deep subcutaneous and visceral rather than ‘safe’ superficial subcutaneous adipose compartments (16) that are proposed to contribute to an increased risk of developing T2D. Even in outwardly lean individuals, increased visceral adiposity and associated ‘lipid overspill’ into ectopic non-adipose tissue organs, such as pancreas and liver, alters normal physiological control worsening insulin resistance and pancreatic *β*-cell dysfunction (17). This adverse partitioning of abdominal adipose tissue in favour of visceral and ectopic sites is shown to typify Asian populations compared to other more metabolically resilient cohorts, including for example European Caucasians. This has been termed the Thin-on-the-Outside-Fat-on-the-Inside (TOFI) phenotype (18–21), in part considered a causal driver of increased T2D prevalence (11, 22, 23).

Findings from TOFI_Asia, a cross-sectional observational study previously conducted in our laboratory (7, 10), underscored greater visceral adipose tissue (VAT), ectopic fat and clinical markers (triglycerides, amylin, C-peptide, glucagon), as predictive of increased T2D risk in Asian Chinese compared to age and body mass index (BMI) matched European Caucasians. More importantly, plasma metabolomics revealed a novel and distinct metabolite signature for VAT and dysglycaemia between the two ethnic groups (8). These differences included amino acid-related metabolites involved in tryptophan and histidine metabolism, the methyl transfer pathway, sugar derivatives, gut microbial metabolites, exogenous compounds, and lipid species encompassing 15 lipid subclasses. Notably, the causal underpinning of increased metabolic risk and dichotomy in the metabolome could not be determined from the TOFI_Asia observational study. We hypothesise that the observed dichotomy in plasma metabolome may be attributed to 1 or more of 3 key factors: ethnicity (genetic background), pathology (dysglycaemia) and/or lifestyle (habitual diet). To resolve this question, a fully-controlled dietary intervention with associated in-depth multi-omic investigations is required.

The importance of diet in preventing T2D progression, through its effect on body weight and metabolic control, is unequivocal with several renowned dietary patterns (24–27) aligned with the World Health Organisation Global Action Plan for the Prevention and Control of Noncommunicable Diseases (28). An exemplar is the Mediterranean Diet (MD), adapted for different populations (29, 30) including Asia (31–34) and more recently New Zealand (35) in accordance with guidance provided by the New Zealand Ministry of Health (NZ MoH) (36). Of note, the latter was a community-based trial conducted in individuals with increased cardiometabolic risk (metabolic syndrome severity score: MetSSS>3.5), with a food basket of healthy items provided to participants and their families at home, and therefore without strict dietary control (35). Despite evidence supporting dietary and lifestyle interventions as effective for amelioration of T2D risk in prediabetes, these positive effects are commonly driven by concomitant weight loss (34). However, whether diet modification in the absence of weight loss can be successful for T2D prevention is far less certain. Notably this is of particular relevance for populations characterized by the TOFI phenotype (37) in whom adverse metabolic outcomes, related to adiposity and ectopic fat infiltration, are evidenced even in the absence of overweight and obesity.

Here we describe a 2-week fully controlled diet intervention study protocol, conducted within a residential facility, under conditions of energy balance (defined as body weight stability), in Asian Chinese and European Caucasian adults with prediabetes. The trial will investigate, firstly, the cause(s) underlying the differences in plasma metabolome observed between ethnicities in the TOFI_Asia study (8) and, secondly, the effect of fully supervised dietary intervention on biomarkers of T2D in at risk populations. The diets to be investigated are a generic current Best Practice Healthy Diet (‘BPHD’) and a New Zealand-specific healthy diet comprising a ‘synergy’ of foods based on principles of the MD, termed the ‘SYNERGY’ diet. Effects of ethnicity will be investigated by a within-diet comparison of Asian Chinese vs. European Caucasian cohorts allocated to BPHD. We hypothesise that 14-days of matched BPHD will result in a diminishing of the previously observed dichotomy between plasma metabolome, with consequent overlap of metabolite features. Effects of lifestyle (habitual diet) on metabolic risk markers will also be investigated by a between-diet comparison of the Chinese cohort randomised to either BPHD or the SYNERGY diet. We hypothesise, firstly, that both diets will improve adverse metabolic markers over 14 days and, secondly, that the SYNERGY diet - adapted using principles of the MD but in a New Zealand setting - will significantly improve risk compared to BPHD.

## 2 Methods and analysis

### 2.1 Trial design and objectives (SPIRIT 7, SPIRIT 8, SPIRIT 17a)

**Figure 1:**
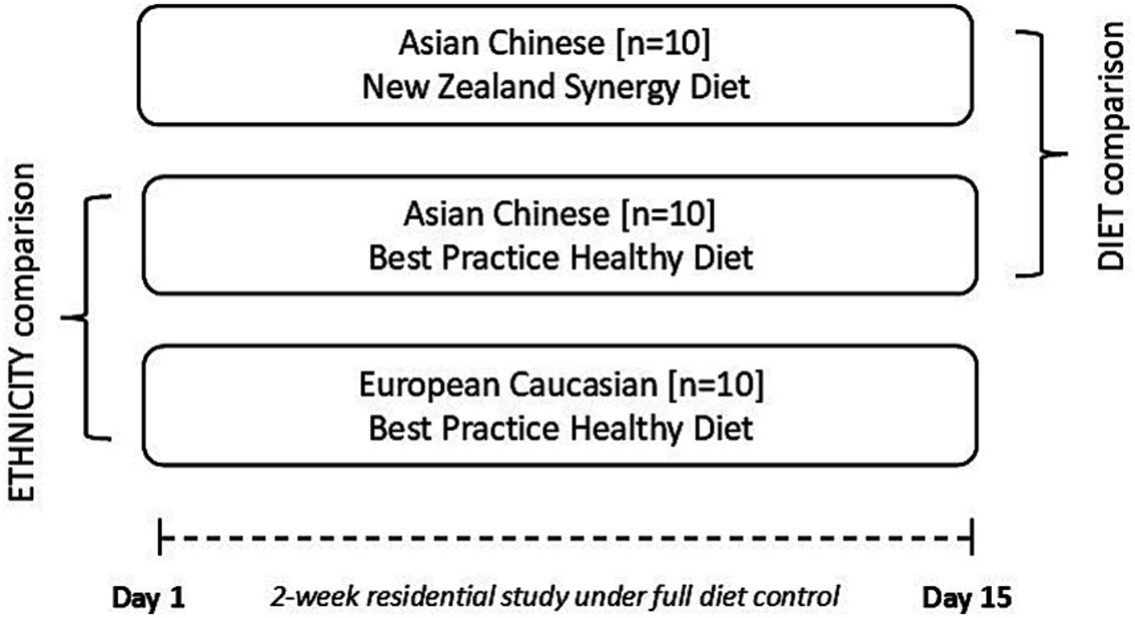
Illustration of the single blind, parallel study design that aims to investigate, in a full dietary controlled residential setting, metabolic improvements following provision of a matched diet (BPHD) in two ethnic groups (within-diet ethnicity comparison – Asian Chinese vs. European Caucasian), and comparator diets, in one ethnic group (between-diet comparison – BPHD vs. SYNERGY).

The trial was conducted as a single-blind, parallel design, residential, fully diet-controlled, 3-arm intervention of 2 weeks duration, in 2 Asian Chinese and 1 European Caucasian cohorts (Figure 1). Two *a priori* hypotheses will be investigated by comparing (1) the effect of ethnicity (ETHNICITY, within-diet comparison) between Asian Chinese and European Caucasian participants allocated to BPHD, and (2) the effect of dietary intervention (DIET, within-ethnicity comparison) between 2 cohorts of Asian Chinese participants randomised to BPHD or SYNERGY.

**Figure 2:**
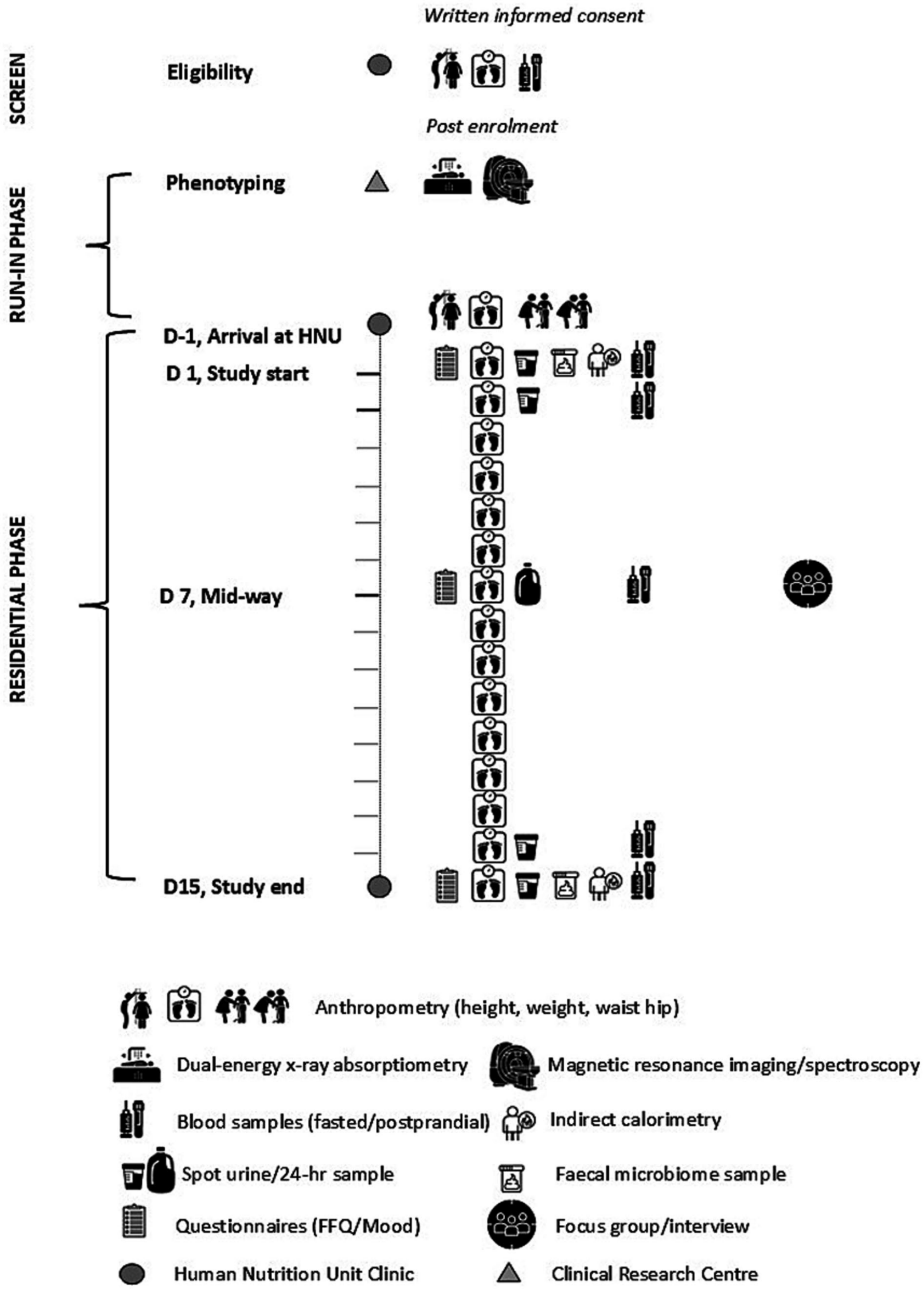
Schematic diagram of the New Zealand SYNERGY Study protocol depicting the assessments conducted during screening, run-in and diet-controlled residential phases of the study, Day (D) -1 to D15, in each of the three intervention arms.

This article follows the SPIRIT (Standard Protocol Items: Recommendations for International Trials) guidelines for reporting clinical trial protocols with the time schedule of enrolment, intervention, and assessments detailed in Table 1 and Figure 2.

**Table 1:** Schedule of enrolment, interventions, and assessments conducted in the New Zealand SYNERGY Study.

	Study period								
	Recruitment/Enrolment		Randomisation (AC)/Allocation (EC)	Post randomisation/allocation					
	Pre-screen (RedCap, telephone)	Screen visit (In person, HNU clinic)		Run-in Phase	Residential Phase Day −1	Residential Phase Day 1	Residential Phase Day 7	Residential Phase Day 14	Residential Phase Day 15
Timepoint (t)	-t_2_	-t_1_	t_0_	t_1_	t_2_		t_3_	t_4_	t_5_
Eligibility Screen									
Questionnaire	x ^$^	x							
Informed Consent		x							
Randomisation/ Allocation			x						
Intervention									
Best Practice Healthy Diet (BPHD)						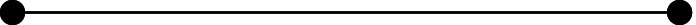			
New Zealand SYNERGY Diet						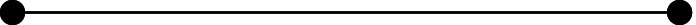			
Assessments									
DXA scan				x					
MRI/S scan				x					
FFQ					x				
Mood Questionnaire						x	x		x
Body weight		x			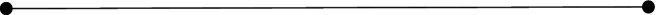				
Height		x			x				
Waist circumference					x				
Hip circumference					x				
Blood pressure		x				x			x
Fasted blood sample* ^#^		x			x	x	x	x	x
Feacal sample						x			x
Urine sample						x	x^†^	x	x
Indirect Calorimetry						x			x
Body temperature						x			x
Heart Rate						x			x
OGTT ^#^						x			x
Focus group/interview							x		

^$^Demographic and contact information, self-reported bodyweight & height, FINDRISC, medications and supplement intake, recent blood donation. ^*#^Analyses of established clinical markers of type 2 diabetes risk; ^*^Plasma metabolomic analyses; ^*#^Immunomodulation; ^†^24-h urine nitrogen. REDCap, Research Electronic Data Capture; HNU, Human Nutrition Unit; AC, Asian Chinese; EC, European Caucasian; FINDRISC, Finnish Diabetes Risk Score; DXA, Dual-Energy X-Ray Absorptiometry; MRI/S, ^*#^Magnetic Resonance Imaging and Spectroscopy; FFQ, Food Frequency Questionnaire; OGTT, Oral Glucose Tolerance Test.

### 2.2 Study setting (SPIRIT 9, SPIRIT 11c)

A fully diet-controlled, residential study was conducted in Mount Eden, Auckland, New Zealand at the Human Nutrition Unit (HNU), University of Auckland (UoA). This is a 5-bed residential metabolic facility unique within New Zealand and one of few such facilities worldwide. All meals were prepared as individual servings at the HNU by a metabolic cook and personalised for individual participants with respect to energy (MJ) content in order to maintain energy balance and body weight stability over the 2-week intervention. Participants were provided with all meals and snacks, comprising 3 main meals [breakfast, lunch, dinner] and 2 snacks [mid-morning, mid-evening], plus 1.5 L of Chinese-style tea per day. All food items provided were required to be consumed in their entirety, to maximise compliance, before 10 pm each day. No other foods or beverages were allowed. The compliance threshold for exclusion from the trial was set at 100 +/− 5%. Participants were identified as non-compliant if intake on any single day of the trial dropped below 95% (study foods not consumed) or increased above 105% (non-study foods consumed). Two main meals, breakfast and dinner, were consumed in the HNU dining room under supervision, to ensure 100% compliance. This was determined by visual confirmation by research staff, with the requirement that all served food was consumed in its totality. The lunch meal and snacks were packed if required, to ensure no disruptions to the participants’ regular work schedule, and could be consumed at HNU or at work. Participants were, however, required to retain the empty food packaging in their snack bags and return them to the HNU staff daily upon arrival, to monitor adherence and ensure full diet compliance. In addition, nitrogen balance (dietary N_in_ – urine N_out_) was assessed during the 2 weeks. To eliminate bias and confounding participants were requested not to e-share or upload photographs or other information about the diets or the study protocol to social media, friends and family over the study period.

**Figure 3:**
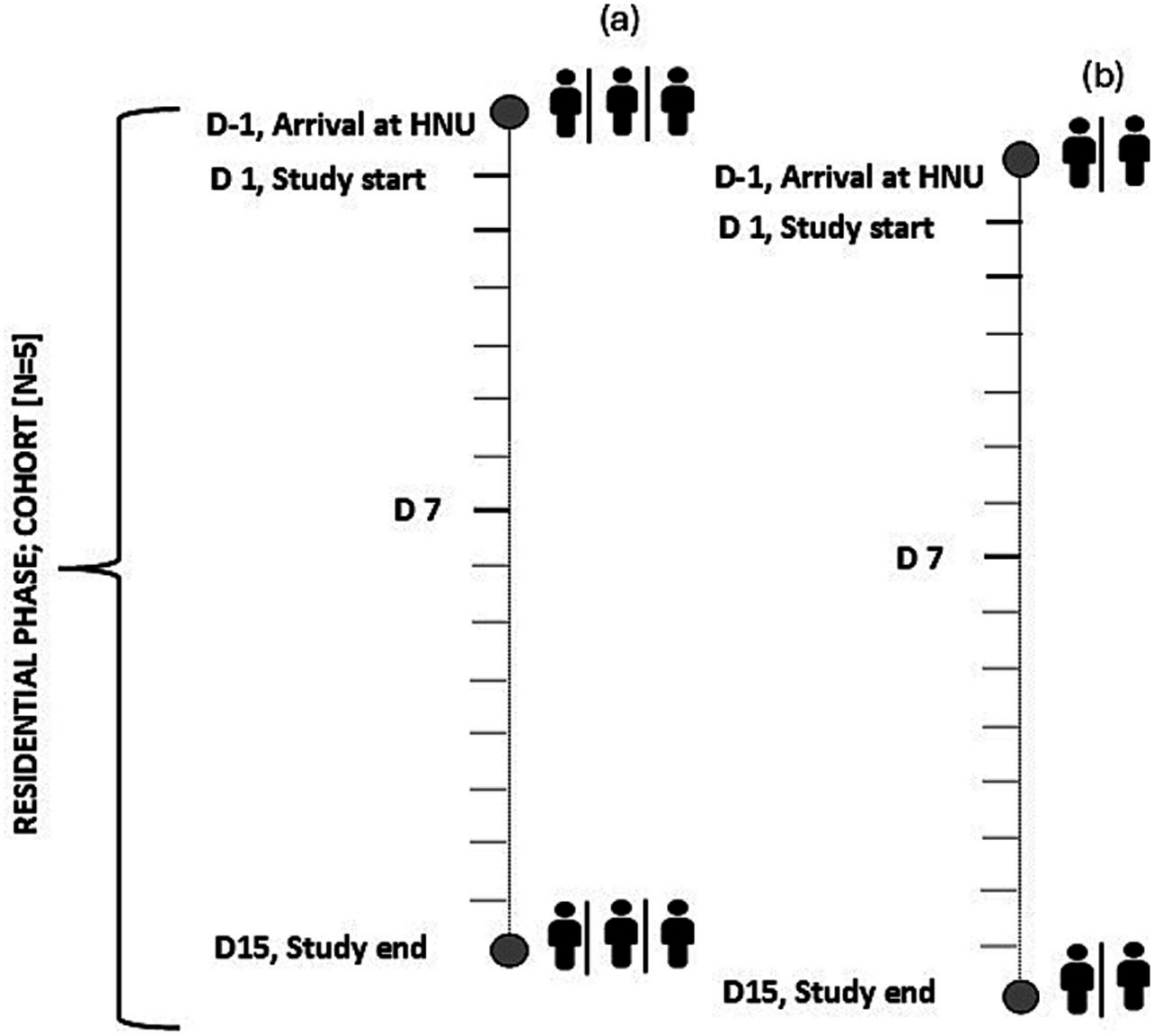
Schematic of the planned staggered start for each cohort of *n* = 5 (sub-group *n* = 3, *n* = 2) participants resident at the Human Nutrition Unit over the 2-week residential study. D, Day; HNU, Human Nutrition Unit.

Cohorts of 5 participants within the same randomised diet group were planned (i.e., 6 cohorts in total), with a 1-day staggered start between participants 1, 2, 3 (sub-group a) and participants 4, 5 (sub-group b, Figure 3). Thus, ensuring careful monitoring by research staff and maximised compliance throughout the 14-day intervention. Deviations from the dietary protocol and/or adherence to instructions provided would be considered as non-compliance and reviewed by the Principal Investigators.

Phenotyping assessments of body composition comprising dual-energy X-ray absorptiometry (DXA) and magnetic resonance imaging and spectroscopy (MRI/S) were conducted at the Clinical Research Centre and at the Centre for Advanced MRI respectively, in the Faculty of Medical and Health Sciences, UoA. As the study aimed to assess metabolic changes under conditions of weight stability, i.e., no weight loss, participants were requested to maintain their typical daily activities with strict avoidance of any vigorous physical activity that involved high-intensity exercise, strength training or sports.

### 2.3 Eligibility and recruitment (SPIRIT 10, SPIRIT 15)

Participants were included in the study if they were 18–60 years of age, self-identified as European Caucasian or Asian Chinese (including mainland China, Singapore, Malaysia, Hong Kong, Taiwan) (38), and had BMI in the overweight-obese range (based on ethnic-specific cut points; 24–40 kg/m^2^) (39) and fasting plasma glucose (FPG) in the prediabetic range, 5.6–6.9 mmol/L (40). Participants self-reported as healthy and willing to participate in a 2-week residential study. Exclusion criteria were significant prior weight gain or loss (>10%) in the previous 3 months, bariatric surgery, current or prior medical history of relevance (e.g., diabetes, cardiovascular disease, pancreatic disease, cancer, liver disease, digestive diseases including inflammatory bowel syndrome/disease, ulcerative colitis, Crohn’s disease), current prescription medication affecting body weight or glucose metabolism (e.g., glucocorticoids), smoking and/or recreational drug use in the previous 6 months, pregnancy or breastfeeding, and blood donation in the previous 3 months. Standard exclusions for a dietary intervention study also included any dislike or unwillingness to consume food items in the diets (e.g., animal products) or hypersensitivities or allergies to these foods (e.g., lactose intolerance, nut allergies), an unwillingness to comply with the residential study protocol which included consuming all foods that were provided in their entirety, and participation in prior clinical studies in the previous 6 months.

A rolling recruitment for each residential cohort was adopted between May 2021 to October 2023 with participants recruited from the wider Auckland region using advertisements posted on general and social media. Participants involved in previous clinical studies conducted at the HNU, and who had consented to be contacted for future studies, were also invited to participate. All individuals who expressed interest in the residential study were invited to contact the HNU for written information on the study. Data on gender, age, ethnicity, reported body weight and height, diabetes risk as determined by FINDRISC (Finnish Diabetes Risk Score) (41), brief medical history, current medications, supplement intake were collected using the Research Electronic Data Capture (REDCap) platform hosted at the UoA (42, 43), and via telephone. This pre-screen ensured that inclusion/exclusion criteria were likely to be met prior to attending in-person screening at the HNU clinic (Table 1; Figure 2). Participants were requested to bring prescriptions or a list of current medications from their primary health care doctor/general practitioner (GP) to the screening visit.

### 2.4 Consent and randomisation (SPIRIT 16a & c, SPIRIT 26a)

All participants received a participant information sheet-informed consent form (PIS-ICF) detailing the study protocol, and all provided written informed consent prior to data collection. Translations of the PIS-ICF were provided for Asian Chinese participants upon request. On the morning of the scheduled day, participants attended in-clinic screening following an overnight fast. All queries regarding study assessments and procedures were addressed by the research team, with Mandarin and Cantonese translation as required, prior to participants providing written informed consent. They were reminded that any supplement intake would need to be discontinued, and only light activity to be undertaken over the 14-day study period. Pre-screening data were then checked, and questionnaires completed. These included a food preference questionnaire to determine aversions and/or allergies to food items in the study diets. Participants were then presented with an example menu to review and, where requested, also provided with a sample of the prepared food items to taste. Anthropometry was recorded and a fasted venous blood sample collected to determine prediabetes status. All blood collections were undertaken before 10:30 am to avoid circadian variability.

Following confirmation of eligibility, European Caucasian participants (*n* = 10) were allocated to receive BPHD, and Asian Chinese participants (*n* = 20) were randomly allocated at a 1:1 ratio to receive either BPHD or SYNERGY, using an online randomisation tool.^1^ Participants were enrolled into ethnicity-diet group cohorts (maximum *n* = 5), each of which were allocated a predetermined randomised start date. This ensured temporal or seasonal effects that may act as confounders over the study period were minimised, and also accounted for the maximum bed capacity at the HNU clinic. Hence eligible participants were randomly allocated to either early or late phase cohorts (Table 2) and started the residential study in a predetermined order. The study was conducted over 2 periods of country-wide New Zealand COVID-19 shut-down, and an Auckland flood and cyclone, which required the predetermined order of randomisation to be amended to mitigate COVID risk and associated drop in recruitment during this phase. The initial plan of 6 cohorts of *n* = 5 participants (Table 2) was expanded to 16 cohorts of fewer participants, with the intervention conducted between May 2021 and November 2023.

**Table 2:** Planned conduct of pre-determined and randomised ethnicity-diet group cohorts.

Cohort	Ethnicity	Diet arm^#^	*n*
1 [early start]	European Caucasian*	Best Practice Healthy Diet	5
2 [early start]	Asian Chinese**	Best Practice Healthy Diet	5
3 [early start]	Asian Chinese**	New Zealand SYNERGY Diet	5
4 [late start]	European Caucasian*	Best Practice Healthy Diet	5
5 [late start]	Asian Chinese**	Best Practice Healthy Diet	5
6 [late start]	Asian Chinese**	New Zealand SYNERGY Diet	5

^#^Predetermined order to be maintained throughout the study period, ^*^European Caucasian participants randomly allocated to Cohort 1 (early start) or Cohort 4 (late start). ^**^Asian Chinese participants randomised to either New Zealand SYNERGY diet or Best Practice Healthy Diet (i.e., trial randomisation). Then randomly allocated to Cohort 2 or 5 for Best Practice Healthy Diet, or Cohort 3 or 6 for SYNERGY.

**Figure 4:**
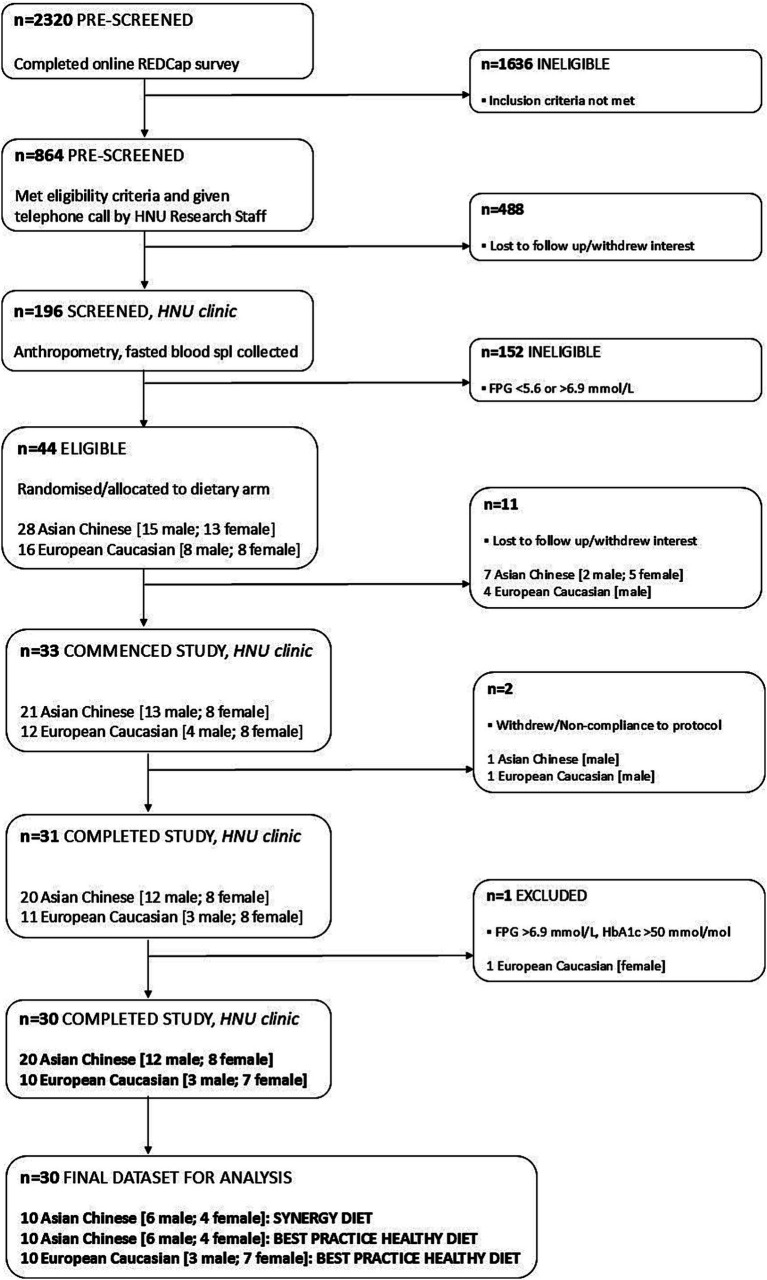
Flowchart illustrating enrolment and eligibility of participants into the 2-week residential study.

As detailed in the CONSORT flow diagram (Figure 4), a total of 2,320 volunteers were pre-screened for eligibility, of which 196 participants attended in-person screening at the HNU clinic. Forty-four participants met the eligibility criteria and were enrolled into the study with 31 participants (*n* = 20, Asian Chinese; *n* = 11, European Caucasian) undergoing all assessments and completing the 2-week residential study. One European Caucasian participant was later identified with raised FPG and HbA1c within the diabetic range (40) and was excluded. A total of 30 participants [*n* = 10 Asian Chinese, SYNERGY; *n* = 10 Asian Chinese, BHPD; *n* = 10 European Caucasian, BPHD] completed the trial and will be included in the analyses. Two participants were excluded from the study during the intervention due to non-compliance to diet; *n* = 1 (Asian Chinese male; BHPD) excluded on day 4 – unwilling/unable to consume multiple food items, and *n* = 1 (European Caucasian male; BHPD) excluded on day 8 – unwilling/unable to consume all foods items.

### 2.5 Diet groups (SPIRIT 11a & b)

The dietary intervention contained both animal and plant products, incorporated elements of a typical Asian Chinese-style diet, and was approved by a NZ Registered Dietitian. Accordingly, both BPHD and SYNERGY adhered to the NZ MoH (36) and Chinese National dietary guidelines (44) for optimising metabolic health. SYNERGY was designed to incorporate the principles of the MD whilst featuring a combination of 20 nutritional products, provided in-kind by 13 New Zealand Food and Beverage companies (Table 3). These products included those enriched with a combination of protein, omega-3 fatty acids and dairy-origin ingredients; low glycaemic index (GI) complex carbohydrates, fibre, pseudo cereals and fruits; polyphenol and antioxidant rich whole-fruit powders and oolong tea. Products were selected based on an established evidence-base to support enhanced benefit to glycaemic/metabolic health (28). Both BPHD and SYNERGY diets were designed using FoodWorks 10 Professional (v10.0, Xyris Pty Ltd., Qld, Australia) with matched energy content. Recipes were developed and adapted to include novel ingredient replacements as differentiators between the 2 diets, as shown in Supplementary Table 1.

**Table 3:** High quality nutritional products, provided by New Zealand food and beverage companies, incorporated into the SYNERGY diet.

	Company	Product
1	Anagenix Limited	Feiolix®
2	Ārepa	Nootropic Powder
		Neuroberry®
		Performance drink
3	Ceres Organics	Rice medley
		Black rice
4	Fonterra Co Operative Group Limited	Anchor Calci+™ Trim Milk
		Anchor Protein+™ Yoghurt
5	Harraway & Sons Limited	Harraways Oat-Activ® [Quick Cook Style Rolled Oats with (4.5% of recipe mix) plant sterol’s]
		Harraways Traditional Rolled Oats
6	LUMINA®	Lamb
7	Mt Cook Alpine Salmon Limited	Salmon
8	NUKU ki te Puku™	Nut bar, prototype product*
9	Sanitarium Health Food Company	Weet-Bix™ Hi-Bran
		Weet-Bix™ Multi-Grain
		Up&Go™
10	The New Zealand Quinoa Co	Quinoa Puffs
		Quinoa
11	T&G Fresh Limited	Lotatoes™
12	Zealong Tea Estate Limited	Oolong tea^$^
13	Zespri™ International Limited	SunGold™ kiwifruit

^*^Nutrients. 2024; 16 (13): 210; ^$^caffeine: 0.09 mg/mL.

All meals planned within each dietary arm were provided as a 5-day rotating menu over the 2-week study period, such that Menu Days 1–5 were repeated on days 6–10 and again on days 11–14. Daily energy intake was individualised to match each participant’s estimated daily energy requirements with body weight monitored daily. Requirements were calculated using the Harris-Benedict equation (45), and a physical activity level (PAL) constant of 1.4, equivalent to sedentarity. Worked examples of both diets, based on 10 MJ intake per day, are shown in Table 4. Accordingly, on average over the 5-day rotating menu plan, BPHD comprised 57 en% total carbohydrate with 8.2 en% added sugar and 32.9 g fibre; 25 en% total fat with 7 en% saturated fat; and 15 en% protein. In contrast, SYNERGY comprised significantly lower total carbohydrate (46 en%, *p* < 0.001) with significantly lower added sugar (1.8 en%, *p* = 0.008) and significantly higher fibre (45.1 g, p = 0.008); significantly higher total fat (30 en%, *p* = 0.03) with similar 7 en% saturated fat, but significantly higher monounsaturated fatty acids (MUFA: 9.7 en%, *p* = 0.01) and polyunsaturated fatty acids (PUFA:5.4 en%, *p* = 0.004); and significantly higher protein (21 en%, *p* < 0.001). All meals were prepared on an individual basis for each participant in 0.5 MJ increments based on energy requirements for body weight maintenance. E.g. in a male participant where calculated total daily energy expenditure was 9.8 MJ, BMR 7.0 MJ and PAL 1.4, a 10 MJ/day diet was allocated. A consistent increase or decrease in body weight over 3-days was a trigger to increase or decrease the daily intake by 0.5 MJ.

**Table 4:** Average macronutrient profile provided by BPHD and SYNERGY diet, over the 5-day menu rotation, based on calculated energy requirement of 10 MJ/d.

Nutrient	BHPD	SYNERGY	*p*-value
Energy (MJ/d)	10.0 ± 12.8 (10.0–10.0)	10.1 ± 83.1 (10.0–10.2)	0.30
Protein (g/d)	86.5 ± 2.2 (83.7–89.3)	122.7 ± 5.1 (116.3–129.0)	**<0.001**
Protein (% en/d)	14.7 ± 0.4 (14.2–15.1)	20.7 ± 0.8 (19.7–21. 7)	**<0.001**
Carbohydrate (g/d)	343.6 ± 18.5 (320.7–366.6)	275.4 ± 18.8 (252.0–298.8)	**<0.001**
Carbohydrate (% en/d)	57.1 ± 3.0 (53.4–60.8)	45.6 ± 3.2 (41.7–49.6)	**<0.001**
Starch (g/d)	226.8 ± 18.5 (203.8–249.7)	182.7 ± 9.3 (171.2–194.2)	**0.003**
Starch (% en/d)	37.8 ± 3.1 (34.0–41.6)	30.3 ± 1.7 (28.3–32.4)	**0.003**
Total Sugar (g/d)	116.9 ± 18.9 (93.5–140.3)	92.7 ± 20.0 (67.8–117.6)	0.09
Total Sugar (% en/d)	19.5 ± 3.1 (15.6–23.4)	15.4 ± 3.4 (11.2–19.6)	0.08
Glucose, Sucrose, Maltose (g/d)	36.8 ± 5.8 (29.5–44.0)	32.9 ± 6.3 (25.0–40.7)	0.34
Glucose, Sucrose, Maltose (% en/d)	6.1 ± 1.0 (4.9–7.3)	5.5 ± 1.1 (4.1–6.8)	0.33
Fructose, Lactose (g/d)	29.5 ± 5.1 (23.2–35.9)	41.7 ± 13.4 (25.0–58.4)	0.12
Fructose, Lactose (% en/d)	4.9 ± 0.9 (3.9–6.0)	6.9 ± 2.2 (4.1–9.7)	0.12
Added sugar (g/d)	48.9 ± 17.7 (27.0–70.8)	10.8 ± 1.4 (9.2–12.5)	**0.008**
Added sugar (% en/d)	8.2 ± 2.9 (4.5–11.8)	1.8 ± 0.2 (1.5–2.1)	**0.008**
Added sugar:total sugar (%)	40.0 ± 12.2 (24.8–55.2)	12.1 ± 3.1 (8.3–16.0)	**0.005**
Fibre (g/d)	32.9 ± 5.4 (26.2–39.5)	45.1 ± 5.6 (38.1–52.0)	**0.008**
Fat (g/d)	66.3 ± 8.2 (56.2–76.5)	78.7 ± 6.2 (71.0–86.4)	**0.03**
Fat (% en/d)	24.7 ± 3.4 (20.5–28.9)	29.5 ± 2.2 (26.7–32.3)	**0.03**
Saturated fat (g/d)	18.9 ± 3.5 (14.6–23.2)	18.1 ± 3.1 (14.3–22.0)	0.72
Saturated fat (% en/d)	7.0 ± 1.3 (5.4–8.6)	6.8 ± 1.2 (5.4–8.2)	0.79
MUFA (g/d)	14.2 ± 1.8 (11.9–16.5)	26.0 ± 6.2 (18.3–33.7)	**0.01**
MUFA (% en/d)	5.3 ± 0.7 (4.5–6.2)	9.7 ± 2.3 (6.9–12.6)	**0.01**
PUFA (g/d)	6.1 ± 3.1 (2.3–9.9)	14.4 ± 3.4 (10.2–18.6)	**0.004**
PUFA (% en/d)	2.3 ± 1.2 (0.8–3.7)	5.4 ± 1.3 (3.8–7.0)	**0.004**
PUFA: MUFA	0.4 ± 0.2 (0.2–0.6)	0.6 ± 0.0 (0.5–0.6)	0.12
Total omega-3 fatty acids (g/d)	0.7 ± 0.4 (0.2–1.2)	1.8 ± 0.9 (0.7–2.9)	0.05
Plant omega-3 fatty acids (g/d)	0.6 ± 0.4 (0.1–1.1)	1.4 ± 0.6 (0.6–2.1)	0.06
Marine omega-3 fatty acids (g/d)	0.04 ± 0.1 (−0.03–0.1)	0.4 ± 0.4 (−0.06–0.9)	0.09

Data presented are Mean ± SD (95% Confidence Interval). Level of significance set at *p* < 0.05. MJ/d, megajoule/day; g/d, gram/day; % en/d, percentage energy/day; MUFA, monounsaturated fatty acids; PUFA, polyunsaturated fatty acids.

### 2.6 Study protocol (SPIRIT 13)

#### 2.6.1 Run-in phase

All participants upon enrolment into the study underwent deep phenotyping. Body composition was determined in the fasted state using DXA, with abdominal fat and ectopic pancreas and liver fat determined using MRI/S, within a fortnight prior to the start of the residential phase (Figure 2). Participants underwent prior health and safety monitoring for contraindications for MRI/S and were provided with instructions for adherence to scan protocol and imaging requirements. A faecal microbiome collection kit was dispensed. A food frequency questionnaire (FFQ) to determine habitual dietary intake, with detailed written protocol for completion, was also dispensed. Participants were instructed to bring these items to the HNU clinic on Day −1 of the residential phase (Figure 2).

#### 2.6.2 Residential phase

Participants arrived at the HNU clinic at half hour intervals on the afternoon of Day −1. They were assigned a bedroom and provided with a Health and Safety pack for residents of the HNU clinic, plus details of assessments scheduled over the study period. Any change in health information including medication since screening was recorded and current phase of the menstrual cycle phase recorded where relevant. Faecal samples were collected and stored at −80°C until batch analyses. Anthropometry was recorded, and a non-fasted venous blood sample (Figure 2) was collected. A visual analogue scale (VAS) mood questionnaire to be completed prior to early the next morning (Day 1) was dispensed. Participants were requested to refrain from vigorous physical activity (e.g., running, swimming, biking, team sports) over the 2-week study. A standardised Day −1 evening dinner meal with a fixed ratio of fat:carbohydrate [%en fat:%en carbohydrate: 0.3–0.4] was served at 6.00 pm; matched to the Day 14 evening dinner meal in order to minimise influence of the evening meal on metabolic assessments (46) conducted on the following mornings of Day 1 and Day 15, respectively.

On Day 1 at 6.00 am participants voided their bladder, and a spot urine sample was collected. Fasted body weight was recorded. 250 mL warm water was then consumed to better facilitate blood sampling. Completed VAS mood questionnaires were collected, and participants were prepared for indirect calorimetry (IC) measurements and a 2-h oral glucose tolerance test (OGTT). Start times were staggered by 15–20 min between participants. Following venous cannulation, a fasted blood sample was collected (T-60 min), body temperature (tympanic and skin) and blood pressure (BP) recorded, and resting metabolic rate (RMR) measured using IC. Post stable 30 min RMR measurements, a fasted blood sample (T-5 min) was collected prior to the start of the OGTT. A 75 g glucose drink (GTD75/C, Carbotest, Milton Adams, Akl, NZ) was then provided to participants, which they consumed within 5 min (T0 min). Thereafter, IC was restarted to determine postprandial glucose induced thermogenesis (GIT), with concurrent blood samples collected over the 2-h period (T15, 30, 60, 90, 120 min) of the OGTT. Upon completion, the venous cannula was removed, IC stopped, and body temperature and BP recorded. A timeline detailing these assessments is illustrated in Supplementary Table 2. At 10.00 am participants were served the breakfast meal under supervision in the HNU dining room. The lunch meal and snacks were eaten at 1.00 pm and 4.00 pm, respectively. The dinner meal was eaten under supervision in the HNU clinic dining room at 7.00 pm. In addition, 1.5 L of pre-prepared Chinese-style tea was consumed throughout the day, along with unrestricted tap water.

Body weight was recorded daily upon awakening, after voiding of the bladder (Figure 2) and before the supervised breakfast meal at 7.00 am. Self-reported health was also recorded. On the morning of Day 2, a fasted spot urine and fasted venous blood sample was collected. Again, breakfast was provided at 7.00 am and participants went about their routine activities with subsequent meals/snacks consumed at 10.00 am, 1.00 pm, 4.00 pm and 7.00 pm. At the mid-point of the intervention on the morning of Day 7, a fasted venous blood sample was again collected. A 24-h urine collection kit and VAS mood questionnaire was also dispensed, to be completed by early morning of Day 8. After the dinner meal, participants then took part in a focus group interview (Figure 2; Supplementary Table 2). It is noted that for some cohorts, a one-on-one interview was conducted following withdrawal of other participants (Figure 4). On the morning of Day 14, the protocol for Day 2 was repeated, with a fasted spot urine and fasted venous blood sample collected, and end of study VAS mood questionnaire and faecal collection kit dispensed. On the morning of Day 15, end of study assessments were made as per Day 1, comprising VAS questionnaire, spot urine and venous blood samples, conduct of IC and OGTT, in addition to receipt of the faecal sample (Figure 2). All questionnaires upon return were checked with the participant by research staff for completeness, accuracy and errors.

### 2.7 Assessments and outcome measures (SPIRIT 12, SPIRIT 18)

#### 2.7.1 Anthropometry

Participants were measured lightly clad and without shoes, and body weight and height recorded to the nearest 0.1 kg and 0.1 cm, respectively, using a calibrated digital scale (Mettler Toledo Spider, ZH, Switzerland) and a wall-mounted Stadiometer (Seca 222, HH, Germany). Waist circumference was measured on expiration, midway between the inferior margin of the lower rib and the iliac crest, and hip circumference at the widest point over the greater trochanter, both to the nearest 0.1 cm using an anthropometric tape (Abbott Laboratories, IL, USA). The average of two readings was recorded for each measure.

#### 2.7.2 BP

BP (mmHg) was measured in duplicate, with the participant seated and rested, using an electronic sphygmomanometer (ProBP 2,400, Welch Allyn, NY, USA), with the cuff placed on the non-cannulated arm at least 2.5 cm above the antecubital space, with the arrow on the cuff in line with the brachial artery.

#### 2.7.3 Body composition

Body fat and lean tissue masses were assessed by whole-body DXA (Lunar iDXA, GE Healthcare, WI, USA) according to the manufacturer’s protocols. Total abdominal adipose tissue (AAT) and lean soft tissue masses were determined in a region of interest (ROI) set with a caudal limit at the top of the iliac crest, and height set to 20% of the distance from this limit to the base of the skull (47), with VAT mass automated within the AAT ROI. Subcutaneous adipose tissue will be derived by subtracting total AAT mass from VAT mass obtained from respective DXA algorithms.

#### 2.7.4 Abdominal, visceral, ectopic pancreas and liver fat

Abdominal, visceral, pancreas and liver fat were determined (10) using a 3 T Magnetom Vida Fit MRI scanner (Siemens Healthcare GmbH, Erlangen, Germany). A 3D dual gradient-echo sequence (VIBE) was acquired to separate fat and water signals using a 2-point Dixon technique. Three blocks of 40 × 5 mm axial slices (field of view, FOV, 500 mm x 400 mm, matrix 320 × 256) were acquired using partial Fourier and parallel imaging with total acceleration factor of 3.1. Subsequently, the pancreas was located and imaged to acquire fourteen 5 mm axial slices (FOV 500 × 400 mm, matrix 512 × 410; repetition time/echo times/flip angle/signal averages = 5.82 ms/2.46, 3.69 ms/9o/1) using partial Fourier and parallel imaging with total acceleration factor of 2.8. Localizer images were obtained in the transverse, coronal and sagittal planes and a voxel (2x2x2 cm^3^) placed in the right lobe of the liver. MRS of the selected voxel was acquired using the stimulated-echo acquisition mode sequence with respiratory triggering; echo time: 20 ms, repetition time: 3000 ms and mixing time: 33 ms, 1,024 data points collected with 50 averages. A water-suppressed spectrum with 50 averages was also recorded to detect weak lipid signals. Post scan MR analysis for quantification of visceral and pancreas fat will be undertaken using Image J (48) and spectroscopy for liver fat using SIVIC software (49).

#### 2.7.5 Questionnaires

All questionnaires were translated for Asian Chinese participants where required. Habitual dietary intake was determined using a FFQ adapted from the 2008–2009 New Zealand Adult Nutrition Survey (50) to assess consumption of items within four food groups: ‘Fruit and Vegetables’, ‘Breads and Cereals’, ‘Eggs, Meat, Poultry and Fish’, and ‘Dairy and Alternatives’ over 4-weeks prior to the start of the study. To facilitate completion and lower response burden, the FFQ contained information on standard serving for estimation of energy intake, multiple choice questions for assessment of nutrient intake (e.g., bread type: white, wholemeal, mixed grain, or other), added sugar and fat (e.g., addition of butter or margarine on vegetables), and method of cooking. Quantification of dietary information collected will be undertaken in FoodWorks 10 Professional (v10.0, Xyris Pty Ltd., Qld, Australia).

VAS mood questionnaires (51) comprised a total of sixteen 100-mm lines anchored at either end by antonyms, and three additional lines to determine feelings of stress, anxiety and mental fatigue. Participants were required to mark their responses by placing a vertical line across the 100-mm scale according to their subjective feelings to rate how “alert” (represented by lines anchored by alert–drowsy, attentive–dreamy, lethargic–energetic, muzzy–clearheaded, well-coordinated–clumsy, mentally slow–quick witted, strong–feeble, interested–bored, incompetent–proficient); “calm” (calm–excited, tense–relaxed); and “content” (contented–discontented, troubled–tranquil, happy–sad, antagonistic–friendly, withdrawn–sociable), stressed, anxious and mentally fatigued they felt.

#### 2.7.6 Faecal microbiome

Faecal sub-samples which were collected and stored at −80°C will undergo batch analysis with a cultivation-independent, molecular approach to characterise microbial community taxonomic composition and functional potential. DNA extraction will be undertaken at the School of Biological Sciences, UoA, by using the NucleoSpin DNA Stool kit (Macherey-Nagel, Germany). Extracted DNA will be quantified by Qubit and DNA integrity checked by agarose gel electrophoresis. Shotgun metagenome sequencing (52) will be conducted by a commercial provider (Auckland Genomics Ltd) using NovaSeq 6,000 (Illumina, CA, USA). Additionally, the presence and quantity of ligands for the aryl hydrocarbon receptor (AhR) (53), a ligand-activated transcription factor expressed by a number of immune cells, will be determined using a luciferase reporter assay method (HepG2-Lucia™ AhR cells, InvivoGen) at the Malaghan Institute of Medical Research, Wellington, New Zealand.

#### 2.7.7 Resting and postprandial energy expenditure (EE)

Respiratory gas exchange was measured non-invasively using IC with an open-circuit ventilated hood system (Quark RMR, COSMED srl, Rome, Italy), with gas and turbine calibrations undertaken prior to each scheduled measurement, according to manufacturer’s instructions. The equipment was maintained in a room kept at a constant ambient temperature (20–22°C). Over the experimental period, participants were in a semi-reclined position and instructed to continue their normal breathing pattern and minimise movement to obtain accurate measures of gas exchange. EE (RMR at ~30 min and postprandial GIT at 2 h) and respiratory quotient (RQ) were calculated from the rates of oxygen consumption (VO_2_) and carbon dioxide (VCO_2_) production. Participants were connected to equipment for cardiometabolic monitoring. Tympanic temperature was measured using an ear thermometer (Braun ThermoScan PRO 6000, Welch Allyn, NY, USA). Skin temperature and heart rate were measured continually using a Thermocron iButton (iButton Link Technology, WI, USA) positioned on the fingertip and a wireless chest belt (Polar T34, Finland) respectively, which were both connected to the Omnia software of the IC system.

#### 2.7.8 Focus group/interview

An understanding of perceptions and attitudes in relation to diet and health were determined during focus group sessions/interviews (54) led by a team of experienced qualitative health researchers from Plant & Food Research, with sessions conducted in Mandarin for Asian Chinese cohorts. Additionally, information on thoughts and experience in the study was gathered to help provide information regarding formulation and feasibility of diets designed for prevention of T2D.

#### 2.7.9 Biological samples

Fasted venous blood samples were collected in appropriate BD Vacutainer® blood collection tubes (Becton, Dickinson and Company, NJ, USA) over the study period. Whole blood, plasma and serum were aliquoted and stored at −80°C until batch analysis for determination of established clinical markers of T2D risk, comprising HbA1c, glucose, insulin, C-peptide, glucoregulatory peptides [total amylin, glucagon, total glucagon-like peptide-1 (GLP-1), gastric inhibitory peptide (GIP)], lipid profile and liver enzymes. Clinical markers will be measured at laboratories at the School of Biological Sciences and the Liggins Institute, UoA using internationally accredited methods. Plasma samples will additionally undergo metabolomic analyses (55), to determine novel markers of T2D risk, at AgResearch Ltd., Palmerston North, New Zealand, using a non-targeted mass spectrometry (MS) based approach with lipids and polar metabolites analysed on a Shimadzu Nexera-x2 UHPLC system coupled to Shimadzu LCMS-9030 quadrupole time-of-flight (Q–TOF) MS (Shimadzu Scientific Instruments, MD, USA). Immune profiling will comprise (i) cytokine analyses from serum samples and (ii) phenotypic and metabolic characterisation of peripheral blood mononuclear cells (PBMC) (56), both using spectral flow cytometry (5-laser Aurora, Cytek) at the Malaghan Institute of Medical Research, Wellington, New Zealand. Additional postprandial venous samples were collected during the 2-h OGTT conducted on Day 1 and 15, respectively. Plasma and serum samples were aliquoted and stored at −80°C until batch analyses to determine acute response of clinical markers (glucose, insulin, C-peptide, glucoregulatory peptides, cytokines) to the glucose challenge, and to the dietary intervention over the 2-week period. 24-h urine samples were collected mid-way through the study on Day 7 and will be analysed at an accredited laboratory (Liggins Institute, UoA) as an independent assessment of dietary compliance using the urine nitrogen balance method (57).

The primary outcome for this study is change in plasma metabolome [fasted: Day 1 *vs* Day 15]. The secondary outcomes include change in (i) fasting clinical markers of T2D risk, faecal microbiome, RMR and immune function [fasted: Day 1 *vs* Day 15] and (ii) postprandial clinical markers of T2D risk, GIT and immune function [OGTT: Day 1 *vs* Day 15]. Other outcomes include (i) thoughts and perceptions of diet and health [Day 7] and (ii) 24-h urine nitrogen balance [Day 7].

### 2.8 Power calculations (SPIRIT 14)

*A priori* modelling of sample size was conducted using the lipid and polar metabolite data, from individuals with prediabetes, from the prior TOFI_Asia study conducted by our research group (8). The sample size calculations were based on assumptions required to identify the change in plasma metabolomic biomarker ‘fingerprint’ in response to the 2-week dietary intervention. Using the reported variance in observed plasma metabolomes and estimates of the expected effect size of diet over 2 weeks, the response of the metabolites was modelled to estimate if an effect could be observed with a smaller sample size. Utilising partial least squares-discriminant analysis modelling, it was estimated that n = 20 individuals (10 per group), was required in each of the 2 ethnicity cohorts to detect a significant response [≥0.8 (1-*β*)] in between-group comparisons. Due to small number of participants recruited to the trial, the results are considered exploratory with no adjustment for multiple testing.

### 2.9 Statistical plan (SPIRIT 20a, b & c)

**Figure 5:**
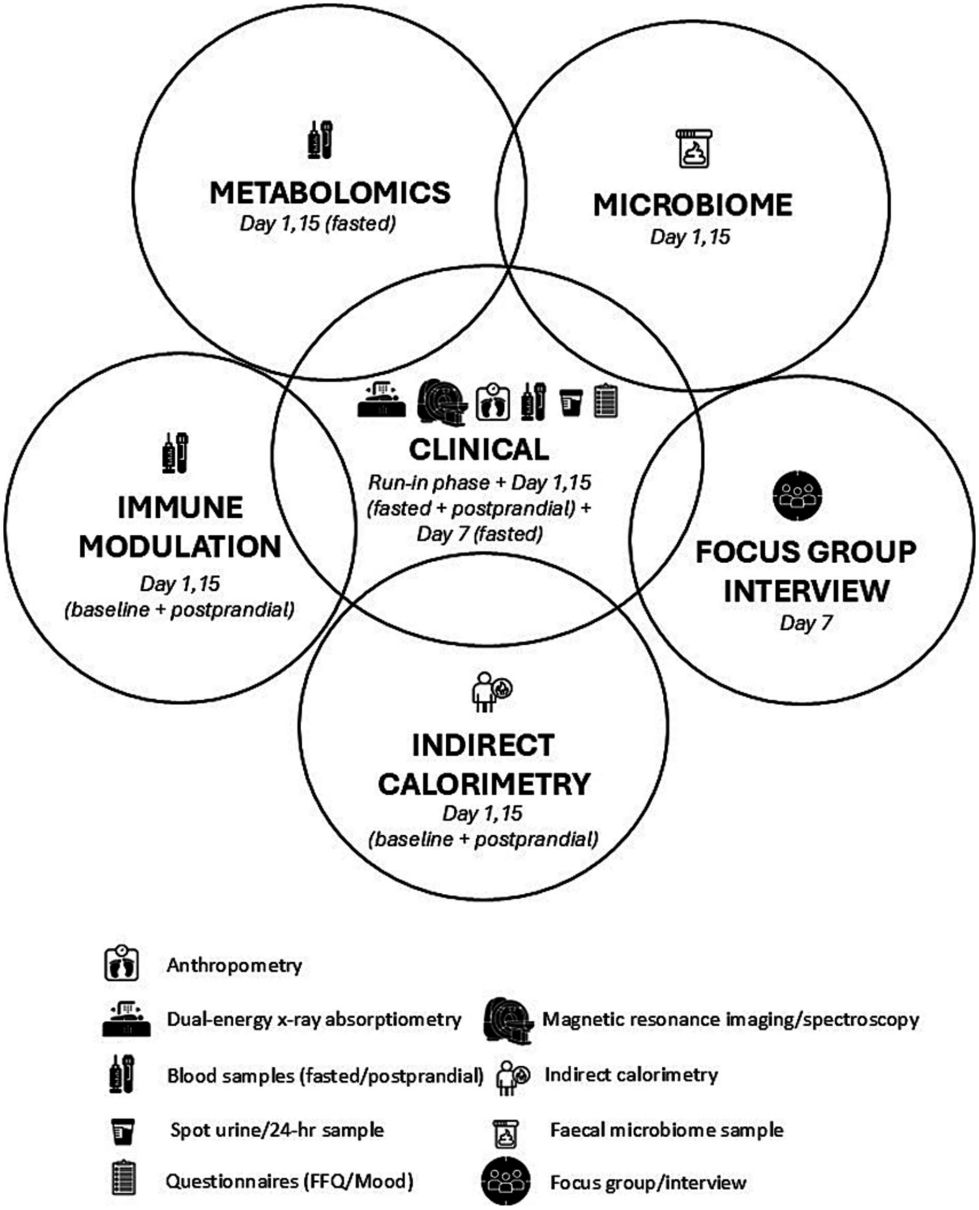
Illustration of the relationship between assessments conducted over the 2-week residential study.

Statistical analyses to test *a priori* hypotheses, related to ethnicity and diet, will be conducted utilising clinical, metabolomic, microbiome, IC, immune and qualitative survey data (Figure 5) from all Asian Chinese participants randomised to the SYNERGY and BPHD, and European Caucasian participants allocated to BPHD. Descriptive statistics and graphical presentations will be used to summarise participant characteristics of key outcome variables. For continuous variables, mean and standard deviation (SD) will be reported unless otherwise specified. For categorical variables, frequencies and percentages will be reported. Differences between diet and ethnic groups will be tested by fitting regression models suitable to the distribution of the outcome variables (e.g., Analysis of Variance, ANOVA or linear mixed effects model, LMM). Non-normally distributed variables will be log-transformed if deemed appropriate. Baseline covariates will be considered in the model, and a correlation matrix will be used to identify significant interactions between potential confounders. These may include age, gender and components of anthropometry and body composition parameters. Missing data will be reported and excluded from the analysis. Model estimates will be reported on key outcome variables with 95% confidence intervals (CI). Statistical significance will be set at *p* ≤ 0.05, and data analysis will be performed using SPSS Statistics for Windows (v 29.0.2.0; IBM Corp, IL, USA).

Untargeted metabolomics peak detection and annotation will be done using the MS-DIAL software (58) and the resulting metabolomic statistics will be performed using a combination of Metaboanalyst and SIMCA (Sartorius, Sweden). Shotgun metagenome data will be processed in a non-assembled (reads-based) approach using DIAMOND + MEGAN6 (59) for taxonomic and functional characterisation of the faecal microbiome. Flow cytometry data will be processed using FlowJo10 (Tree Star) and OMIQ (Dotmatics) for PBMC, LEGENDPlex™ cloud-based software for cytokines, and analyzed using Prism 10 (GraphPad).

### 2.10 Data management, confidentiality and access (SPIRIT 19, SPIRIT 27, SPIRIT 29)

At screening and following confirmation of enrolment into the study, participants were assigned a unique study-specific identifier (de-identified number) that was used on all data collection documentation. All paper-based documentation will be kept for 5 years by researchers in a secure and locked cabinet at the HNU. All electronic data collection and documentation is password protected, stored on secure University servers and can only be accessed by the principal investigators and delegated research personnel. De-identified conversations that were audio recorded (and later transcribed) during the focus group/interviews, are kept securely at Plant & Food Research (D. M. C). Any quotes gathered during the focus group/interviews do not contain details that could personally identify participants. Only collective thoughts and ideas will be used for research purposes. All biological samples (blood, urine and faeces) were labelled with de-identified study IDs and stored in secure −80°C freezers at UoA. They will be transported to laboratories for analyses in the de-identified form. All laboratories (the HNU clinic and School of Biological Sciences at UoA, the MS facility of AgResearch Ltd., and the Malaghan Institute of Medical Research) are Good Laboratory Practice compliant and have access restricted to staff directly involved in the analyses.

### 2.11 Monitoring (SPIRIT 21a)

An internal data safety monitoring plan, approved by the New Zealand Health and Disabilities Ethics Committee, was instituted as the study did not involve significant safety concerns, risks or complexity. Accordingly, all cannulation and blood draws were managed and overseen by a qualified HNU research nurse. Adverse events were recorded by the research staff and nurse by observing and interviewing participants during the experimental procedures, and over the 2-week study period. The record, on Case Report Forms (CRF), contained a description of the event and included the date of onset and remission, was assessed for severity using the protocol severity grading system (mild, moderate or severe) and contained information on the location, relationship to treatment/diet, intervention required if any, and the outcome of the event. The CRF was reviewed within 24-h by the study medical monitor (R. M) with a plan for serious adverse events to be reported to the Ethics Committee if/when required as per Good Clinical Practice (GCP) guidelines (SPIRIT 22). Non-compliance to dietary intake, and/or non-adherence to study protocol, were recorded and reviewed by the Principal Investigators and according to the study eligibility criteria were determined to be reason for exclusion from the study.

## 3 Discussion

To the best of our knowledge, the New Zealand SYNERGY study is the first comprehensive, fully diet-controlled intervention trial utilising a ‘whole of diet’ approach to investigate the amelioration of T2D risk markers in individuals with overweight/obesity and pre-diabetes, in the absence of weight loss, under residential conditions. Provision and strict dietary control of daily food intake over the 2-week period is intended to maximise compliance to the intervention diets, and to ensure causation of outcome can be determined. All meals and snacks will be prepared and provided on a personalised basis for each participant, each based on estimated daily energy requirements, with body weight monitored daily to ensure energy balance is maintained throughout the intervention. It is acknowledged that due to the residential study design, daily energy requirements are calculated using a PAL constant of 1.4, which is equivalent to a sedentary lifestyle. It is anticipated that although there is no formal measurement of physical activity in the study protocol, persistent non-compliance with respect to vigorous activity may result in change in body weight, which would be identified and the behaviour of the participant reviewed. Moreover, the novelty of the study lies in the ‘whole of diet’ comparison between a generic current Best Practice Healthy Diet (‘BPHD’) and the SYNERGY diet using the principles of the MD but incorporating New Zealand specific food and beverages.

The trial aims to investigate metabolic perturbations and ethnic-specific biomarkers associated with T2D risk and susceptibility in Asian Chinese and European Caucasian cohorts, with in-depth metabolic investigations encompassing a multi-omics systems approach. The study explores the causal underpinning of increased metabolic risk and the effect of dietary intervention, by utilising a systems biology approach with state-of-the-art methods including plasma metabolomics, faecal microbiome, indirect calorimetry, whole body composition and quantification of non-adipose tissue ectopic fat, immune profiling and behavioural aspects. There will be 2 main lines of investigation. Firstly, to determine the cause of our previously observed distinct and separate plasma metabolome ‘fingerprints’ in these two ethnic groups, and secondly, to validate these biomarkers as sensitive to dietary intervention. Specifically, the intervention will determine the efficacy of BPHD and the NZ-specific SYNERGY diet for T2D risk amelioration under conditions of energy balance and in the absence of bodyweight loss. Importantly, the full diet control will remove background diet fluctuations and maximise compliance to dietary intervention groups, in order to establish a direct cause and effect relationship between diet and biomarkers. As we understand it, this novel study will be the first to detect ethnic-specific dietary responses in a fully-controlled residential setting using a comprehensive multi-omics approach. Ergo, to minimise drop-out and maximise adherence to the study protocol, within a tightly controlled residential setting, a 2-week intervention period was considered optimal. The trial was originally powered to identify significant changes in plasma metabolomic biomarker ‘fingerprint’ (primary endpoint) in response to the 2-week intervention period. It therefore must be noted that the small group size and short duration may limit the detection of the response of other metabolic end points. However, the exploratory findings are of importance as they will further inform the design of larger ‘free-living’ community interventions and, moreover, explore the feasibility of use of these carefully designed diets within the community.

## Data Availability

The datasets generated from this study will be restricted in line with ethical approvals. Requests to access the datasets should be directed to Jennifer Miles-Chan (j.miles-chan@auckland.ac.nz). Individual participant data will not be made available. This is in accordance with National Health and Disability Ethics Committees application that all data generated will only be used for the purpose of this study.

## Footnotes

^1^ http://www.randomization.com

## Glossary

AAT: Abdominal adipose tissue
AhR: Aryl hydrocarbon receptor
ANOVA: Analysis of Variance
BMI: Body mass index
BMR: Basal metabolic rate
BP: blood pressure
BPHD: Best Practice Healthy Diet
CONSORT: Consolidated Standards of Reporting Trials
DXA: Dual-energy x-ray absorptiometry
EE: Energy expenditure
FINDRISC: Finnish Diabetes Risk Score
FFQ: Food Frequency Questionnaire
FPG: Fasting plasma glucose
GCP: Good clinical practice
GIP: Gastric inhibitory peptide
GIT: Glucose induced thermogenesis
GLP-1: Glucagon-like peptide-1
GP: General Practitioner
HNU: Human Nutrition Unit
IC: Indirect calorimetry
LMM: Linear mixed effects model
MD: Mediterranean Diet
MetSSS: Metabolic syndrome severity score
MRI/S: Magnetic resonance imaging and spectroscopy
MS: Mass spectrometry
MUFA: monounsaturated fatty acid
NZ MoH: New Zealand Ministry of Health
OGTT: Oral glucose tolerance test
PAL: Physical activity level
PBMC: Peripheral blood mononuclear cells
PIS-ICF: Participant information sheet – informed consent form
PUFA: polyunsaturated fatty acid
Q–TOF: Quadrupole time-of-flight
REDCap: Research Electronic Data Capture
RQ: Respiratory quotient
ROI: Region of interest
RMR: Resting metabolic rate
SPIRIT: Standard Protocol Items: Recommendations for International Trials
T2D: Type 2 diabetes
TOFI: Thin-on-the-outside-fat-on-the-inside
UoA: University of Auckland
VAS: Visual analogue scale
VAT: visceral adipose tissue
VCO2: Rate of carbon dioxide production
VO2: Rate of oxygen consumption
