# Supplementary material for: Design and conduct of a full diet-controlled, parallel, 2-week residential trial for diabetes prevention without weight loss in Asian Chinese and European Caucasian adults with prediabetes: the New Zealand SYNERGY study
Source: Front Nutr. 2025 Jun 19;12:1590579. doi: 10.3389/fnut.2025.1590579 (PMC12224437; doi:10.3389/fnut.2025.1590579)
Supplement: Supplementary file 1 [file Table_1.docx]

Supplementary Material

**Supplementary Table 1:** 5-day rotating menu for Best Practice Healthy Diet (BPHD) and New Zealand Synergy Diet (SYNERGY) for a 10 MJ/d intake, demonstrating key ingredient and product exchanges between diets.

|  | **BPHD** | **SYNERGY** |
| --- | --- | --- |
| **DAY 1 – 10MJ** | | |
|  |  |  |
| Breakfast | *Toast with peanut butter, jam and juice*   - Multigrain Bread 111.0 g - Crunchy Peanut Butter 20.0 g - Strawberry Jam 30.0 g - Orange Juice 200.0 g - Banana 100.0 g | *Wheat biscuit, milk and fruit*   - Anchor Calci+™ Trim Milk 250.0 g - Anchor Protein+™ Yoghurt 180.0 g - Sanitarium Weet-Bix™ Hi-Bran 70.0 g - Banana 70.0 g |
|  |  |  |
| Mid-morning snack | *Fruit*   - Apple 170.6 g | *Fruit and drink*   - Zespri™ SunGold™ kiwifruit 161.0 g - Ārepa Performance drink 300.0 g |
|  |  |  |
| Lunch | *Chicken Sandwich*  - Multigrain Bread 74.0 g  - Olive and Avocado Margarine 12.0 g  - Sliced roast chicken 50.0 g  - Lite Mayonnaise 15.0 g  - Carrot 10.0 g  - Salad leaves 6.0 g  - Black pepper 0.2 g | *Chicken Sandwich*  *-* Oat Bread 100.0 g  - Olive and Avocado Margarine 12.0 g  - Sliced roast chicken 50.0 g  - Lite Mayonnaise 15.0 g  - Carrot 10.0 g  - Salad leaves 6.0 g  - Black pepper 0.2 g |
|  |  |  |
| Mid-evening snack | *Muesli bar*  - Yogurt and strawberry bar 63.0 g | *Nut bar*  *-* High Protein Nut bar 50.0 g |
|  |  |  |
| Dinner & Dessert | *Chicken and Cous Cous*  - Chicken breast 60.0 g  - Carrot 75.0 g  - Capsicum 50.0 g  - Green beans 50.0 g  - Mushroom 50.0 g  - Soy Sauce 15.0 g  - Oyster Sauce 15.0 g  - Chilli powder 0.3 g  - Ginger 5.0 g  - Garlic 5.0 g  - Sesame oil 2.0 g  - Couscous 100.0 g  *Oat and date balls*  - Quick Oats 72.0 g  - Crunchy Peanut Butter 15.4 g  - Dates 41.1 g  - Cocoa Powder 7.7 g  - Trim Milk 63.8 g | *Salmon and Quinoa*  *-* Mount Cook Salmon 50.0 g  - Carrot 75.0 g  - Capsicum 50.0 g  - Green beans 50.0 g  - Mushroom 50.0 g  - Soy Sauce 15.0 g  - Oyster Sauce 15.0 g  - Chilli powder 0.3 g  *-* Ginger 5.0 g  - Garlic 5.0 g  - Sesame oil 2.0 g  - Quinoa, NZ Quinoa Co 80.0 g  *Oat and date balls*  *-* Harraways Rolled Oats 35.1 g  - Harraways Oat-Activ® 49.1 g  - Butter 3.5 g  - Dates 38.6 g  - Cocoa Powder 7.0 g  - Anchor Calci+™ Trim Milk 63.3 g  - Protein powder 3.5 g |
|  |  |  |
| Beverage | Pre-prepared barley tea 1.5 L | Pre-prepared Zealong Oolong tea……………… 1.5 L |
|  |  |  |
|  |  |  |
|  |  |  |
| **DAY 2 – 10MJ** | | |
|  |  |  |
| Breakfast | *Granola, milk and fruit*   - Full fat Milk 200.0 g - Almond Granola 100.0 g - Peach 130.0 g - Sunflower seeds 11.0 g - Pumpkin seeds 11.0 g | *Wheat biscuit, milk and fruit*   - Anchor Calci+™ Trim Milk 280.0 g - Sanitarium Weet-Bix™ Multi-Grain 90.0 g - Peach 100.0 g - Ārepa Nootropic Powder 5.0 g |
| Mid-morning snack | *Rice puff bites*  - Air baked rice puff 40.0 g  - Roasted Peanut 12.0 g | *Quinoa puffs*  *-* Quinoa Puffs, NZ Quinoa Co 40.0 g  - Roasted almond 10.0 g  - Roasted peanut 10.0 g |
|  |  |  |
| Lunch | *Tuna sandwich*  *-* Multigrain Bread 74.0 g  - Olive and Avocado Margarine 12.0 g  - Tuna 60.0 g  - Lite Mayonnaise 10.0 g  - Carrot 10.0 g  - Salad leaves 6.0 g  - Black pepper 0.2 g | *Tuna sandwich + drink*  *-* Oat Bread 100.0 g  - Olive and Avocado Margarine 12.0 g  - Tuna 60.0 g  - Lite Mayonnaise 10.0 g  - Carrot 10.0 g  - Salad leaves 6.0 g  - Black pepper 0.2 g  - Sanitarium Up&Go™ 200.0 g |
|  |  |  |
| Mid-evening snack | *Fruit*  *-* Banana 161.0 g | *Fruit*  *-* Zespri™ SunGold™ kiwifruit 161.0 g |
|  |  |  |
| Dinner & Dessert | *Slow cooked beef and rice*  *-* Beef 60.0 g  - Carrot 75.0 g  - Leek 100.0 g  - Mushroom 50.0 g  - Stock cube 6.3 g  - Tomatoes 235.0 g  - Garlic 5.0 g  - Oil 2.0 g  - Chinese 5 Spice 0.5 g  - Salt 1.0 g  - Black Pepper 0.5 g  - Medium grain rice 100.0 g  *Apple clafoutis with salted caramel*  *-* Apple 46.6 g  - Honey 5.1 g  - Butter 4.7 g  - Trim Milk 34.9 g  - Egg 23.3 g  - Plain Flour 34.9 g  - Cinnamon 0.5 g  - Zero sugar salted caramel topping 75.0 g | *Slow cooked lamb and rice*  *-* LUMINA® lamb 60.0 g  - Carrot 75.0 g  - Leek 100.0 g  - Mushroom 50.0 g  - Stock cube 6.3 g  - Tomatoes 235.0 g  - Garlic 5.0 g  - Oil 2.0 g  - Chinese 5 Spice 0.5 g  - Salt 1.0 g  - Black Pepper 0.5 g  - Ceres Organics Rice Medley 100.0 g  *Apple clafoutis with yogurt*  - Apple 67.2 g  - Anchor Calci+™ Trim Milk 27.7 g  - Egg 16.8 g  - Harraways Rolled Oats 16.8 g  - Harraways Oat-Activ® 8.4 g  - Almond meal 8.4 g  - Protein powder 3.4 g  - Cinnamon 0.5 g  - Sliced almond 1.8 g  - Anagenix Feiolix® 1.2 g  - Anchor Protein+™ Yoghurt 180.0 g |
|  |  |  |
| Beverage | Pre-prepared barley tea 1.5 L | Pre-prepared Zealong Oolong tea…………………1.5 L |
| **DAY 3 – 10MJ** | | |
|  |  |  |
| Breakfast | *Steamed bun and fruit*   - Chicken steamed bun 200.0 g - Banana 161.0 g | *Omelette, bread and fruit*   - Oat Bread 100.0 g - Egg whites 200.0 g - Mixed beans 70.0 g - Spring onion 10.0 g - Sweet chilli sauce 5.0 g - Oil 2.0 g - Salt 2.0 g - Zespri™ SunGold™ kiwifruit 100.0 g |
|  |  |  |
| Mid-morning snack | *Vegetable muffin*  *-* Plain Flour 48.5 g  - Eggs 5.4 g  - Trim Milk 67.9 g  - Capsicum 22.6 g  - Onion 22.6 g  - Sweet corn 15.1 g  - Parsley 3.2 g  - Cheese 6.5 g  - Oil 8.1 g  - Chilli powder 0.2 g | *Nut bar*  *-* High Protein Nut bar 50.0 g |
|  |  |  |
| Lunch | *Beef sandwich*  *-* Multigrain Bread 74.0 g  - Olive and Avocado Margarine 12.0 g  - Roast beef 50.0 g  - Whole grain mustard 10.0 g  - Carrot 10.0 g  - Salad leaves 6.0 g  - Black pepper 0.2 g | *Beef sandwich*  *-* Oat Bread 100.0 g  - Olive and Avocado Margarine 12.0 g  - Roast beef 50.0 g  - Whole grain mustard 10.0 g  - Carrot 10.0 g  - Salad leaves 6.0 g  - Black pepper 0.2 g |
|  |  |  |
| Mid-evening snack | *Muesli biscuit bites*  *-* Berry muesli bar 55.0 g  - Soft bake biscuit 50.0 g | *Vegetable muffin*  - Harraways Rolled oats 57.6 g  - Eggs 36.0 g  - Anchor Calci+™ Trim Milk 50.4 g  - Protein powder 1.8 g  - Capsicum 15.1 g  - Onion 15.1 g  - Sweet corn 10.1 g  - Parsley 2.2 g  - Cheese 4.3 g  - Oil 3.6 g  - Baking powder 3.6 g  - Chilli powder 0.1 g |
|  |  |  |
| Dinner & Dessert | *Chicken Udon noodles*  *-* Chicken breast 70.0 g  - Udon Noodles 160.0 g  - Baby corn 40.0 g  - Green beans 50.0 g  - Carrot 50.0 g  - Onion 50.0 g  - Mushroom 50.0 g  - Capsicum 50.0 g  - Ginger 5.0 g  - Garlic 5.0 g  - Oyster Sauce 20.0 g  - Soy Sauce 20.0 g  - Oil 2.0 g  - Sesame seeds 2.7 g  - Black pepper 1.0 g  - Corn flour 3.0 g  *Banana Cake with custard*  *-* Banana 49.4 g  - Plain flour 14.9 g  - Eggs 13.4 g  - Butter 11.1 g  - Trim milk 11.1 g  - Cinnamon 0.1 g  - Vanilla Crème custard mix 9.6 g  - Full fat milk 70.4 g | *Salmon buckwheat noodles*  *-* Mount Cook Salmon 70.0 g  - Buckwheat Noodles 100.0 g  - Baby corn 40.0 g  - Green beans 50.0 g  - Carrot 50.0 g  - Onion 50.0 g  - Mushroom 50.0 g  - Capsicum 50.0 g  - Ginger 5.0 g  - Garlic 5.0 g  - Oyster Sauce 20.0 g  - Soy Sauce 20.0 g  - Oil 2.0 g  - Sesame seeds 2.7 g  - Black pepper 1.0 g  - Corn flour 3.0 g  *Banana Cake with fruit*  *-* Banana 46.8 g  - Harraways Oat-Activ® 5.3 g  - Quinoa, NZ Quinoa Co 2.9 g  - Egg white 11.7 g  - Butter 3.5 g  - Anchor Calci+™ Trim Milk 23.4 g  - Cinnamon 0.1 g  - Baking powder 0.4 g  - Sliced almonds 5.9 g  - Ārepa Neuroberry® 5.0 g  - Zespri™ SunGold™ kiwifruit 61.0 g |
|  |  |  |
| Beverage | Pre-prepared barley tea 1.5 L | Pre-prepared Zealong Oolong tea……………… 1.5 L |
|  |  |  |

| **BHPD SYNERGY** | | |
| --- | --- | --- |
| **DAY 4 – 10MJ** | | |
|  |  |  |
| Breakfast | *Pancake, fruit and milk drink*   - Plain flour 80.0 g - Eggs 57.0 g - Stevia 5.0 g - Oil 1.5 g - Banana 50.0 g - Apple 61.0 g - Strawberry Jam 15.0 g - Drinking Chocolate powder (no sugar)30.0 g - Trim Milk 75.0 g | *Pancake, fruit and milk drink*   - Harraways Oat-Activ® 70.0 g - Eggs 115.0 g - Stevia 5.0 g - Gluten 5.0 g - Oil 1.5 g - Anchor Protein+™ Yoghurt 80.0 g - Banana 70.0 g - Ārepa Neuroberry® 5.0 g - Sanitarium Up&Go™ 250.0 g |
|  |  |  |
| Mid-morning snack | *Muesli bar*  *-* Oat and honey muesli bar 63.0 g | *Fruit*  *-* Zespri™ SunGold™ kiwifruit 161.0 g |
|  |  |  |
| Lunch | *Cheese sandwich & Fruit*  *-* Multigrain Bread 74.0 g  - Olive and Avocado Margarine 12.0 g  - Cheese slices 27.6 g  - Beetroot chutney 15.0 g  - Carrot 10.0 g  - Salad leaves 6.0 g  - Black pepper 0.2 g  - Apple 100.0 g | *Cheese sandwich*  *-* Oat Bread 100.0 g  - Olive and Avocado Margarine 12.0 g  - Cheese slices 27.6 g  - Beetroot chutney 15.0 g  - Carrot 10.0 g  - Salad leaves 6.0 g  - Black pepper 0.2 g |
|  |  |  |
| Mid-evening snack | *Rice puff bites with fruit*  *-* Air baked rice puff 40.0 g  - Roasted Peanut 12.0 g  - Mandarin 86.0 g | *Quinoa puffs*  *-* Quinoa Puffs, NZ Quinoa Co 40.0 g  - Roasted almond 10.0 g |
|  |  |  |
| Dinner & Dessert | *Beef and potato casserole*  *-* Beef mince 100.0 g  - Celery 30.0 g  - Carrot 70.0 g  - Onion 50.0 g  - Peas 40.0 g  - Tomatoes 70.0 g  - Garlic 5.0 g  *-* Ginger 1.5 g  - Oyster Sauce 14.8 g  - Fish sauce 6.8 g  - Star anise 1.5 g  - Cinnamon 1.5 g  - Corn flour 2.0 g  - Potato 250.0 g  - Butter 2.0 g  - Rosemary 1.0 g  - Salt 1.5 g  *Mango Rice pudding*  *-* Creamed rice pudding 200.0 g  - Raisins 25.0 g  - Mango 60.0 g  - Cardamom powder 2.0 g | *Lamb and potato casserole*  *-* LUMINA® lamb 100.0 g  - Celery 30.0 g  - Carrot 70.0 g  - Onion 50.0 g  - Peas 70.0 g  - Tomatoes 70.0 g  - Garlic 5.0 g  - Ginger 1.5 g  - Oyster Sauce 14.8 g  - Fish sauce 6.8 g  - Star anise 1.5 g  - Cinnamon 1.5 g  - Corn flour 2.0 g  - Lotatoes™, T&G 250.0 g  - Butter 2.0 g  - Rosemary 1.0 g  - Salt 1.5 g  *Rice pudding*  *-* Ceres Organics Black rice 70.0 g  - Anchor Calci+™ Trim Milk 300.0 g  - Blueberry 50.0 g  - Sliced almond 10.0 g  - Stevia 8.0 g  - Anagenix Feiolix® 1.2 g |
|  |  |  |
| Beverage | Pre-prepared barley tea 1.5 L | Pre-prepared Zealong Oolong tea…………………1.5 L |
|  |  |  |
|  |  |  |
|  | **BHPD** | **SYNERGY** |
| **DAY 5 – 10MJ** | | |
|  |  |  |
| Breakfast | *Oats and fruit*   - Quick Oats 102.0 g - Raisins 10.0 g - Cinnamon 1.0 g - Apple 50.0 g - Banana 110.8 g | *Wheat biscuit, milk and fruit*   - Anchor Calci+™ Trim Milk 300.0 g - Sanitarium Weet-Bix™ Multi-Grain 90.0 g - Cinnamon 0.1 g - Apple 60.0 g - Anagenix Feiolix® 1.2 g |
| Mid-morning snack | *Vegetable Muffin*   - Plain Flour 48.5 g - Eggs 5.4 g - Trim Milk 67.9 g - Capsicum 22.6 g - Onion 22.6 g - Sweet corn 15.1 g - Parsley 3.2 g - Cheese 6.5 g - Oil 8.1 g - Chilli powder 0.2 g | *Vegetable muffin*   - Harraways Rolled oats 57.6 g - Eggs 36.0 g - Anchor Calci+™ Trim Milk 50.4 g - Protein powder 1.8 g - Capsicum 15.1 g - Onion 15.1 g - Sweet corn 10.1 g - Parsley 2.2 g - Cheese 4.3 g - Oil 3.6 g - Baking powder 3.6 g - Chilli powder 0.1 g |
| Lunch | *Ham sandwich*   - Multigrain Bread 74.0 g - Olive and Avocado Margarine 12.0 g - Sliced ham 60.0 g - Whole grain mustard 15.0 g - Carrot 10.0 g - Salad leaves 6.0 g - Black pepper 0.2 g | *Ham sandwich*   - Oat Bread 100.0 g - Olive and Avocado Margarine 12.0 g - Sliced ham 60.0 g - Whole grain mustard 15.0 g - Carrot 10.0 g - Salad leaves 6.0 g - Black pepper 0.2 g |
|  |  |  |
| Mid-afternoon snack | *Muesli bar*   - Oat and berry muesli bar 64.0 g | *Nut bar*   - High Protein Nut bar 50.0 g |
|  |  |  |
| Dinner & Dessert | *Egg fried rice*   - Eggs 138.0 g - Medium grain rice 90.0 g - Baby corn 40.0 g - Capsicum 50.0 g - Peas 50.0 g - Spring onion 100.0 g - Mushroom 50.0 g - Ginger 5.0 g - Garlic 5.0 g - Chilli powder 0.3 g - Soy Sauce 15.0 g - Oil 2.0 g   *Mixed fruit custard*   - Vanilla Crème custard mix 18.0 g - Full fat milk 132.0 g - Banana 30.0 g - Mandarins 75.0 g - Lychees 75.0 g | *Salmon egg fried rice and quinoa*   - Mount Cook Salmon 70.0 g - Eggs 46.0 g - Ceres Organics Rice medley 40.0 g - Quinoa, NZ Quinoa Co 40.0 g - Baby corn 40.0 g - Capsicum 50.0 g - Peas 50.0 g - Spring onion 100.0 g - Mushroom 50.0 g - Ginger 5.0 g - Garlic 5.0 g - Chilli powder 0.3 g - Soy Sauce 15.0 g - Oil 2.0 g   *Mixed fruit yogurt*   - Anchor Protein+™ Yoghurt 180.0 g - Peach 50.0 g - Ārepa Neuroberry® 5.0 g - Zespri™ SunGold™ kiwifruit 161.0 g |
| Beverage | Pre-prepared barley tea 1.5 L | Pre-prepared Zealong Oolong tea…………………1.5 L |
