# Supplementary material for: Design and conduct of a full diet-controlled, parallel, 2-week residential trial for diabetes prevention without weight loss in Asian Chinese and European Caucasian adults with prediabetes: the New Zealand SYNERGY study
Source: Front Nutr. 2025 Jun 19;12:1590579. doi: 10.3389/fnut.2025.1590579 (PMC12224437; doi:10.3389/fnut.2025.1590579)
Supplement: Supplementary file 2 [file Table_2.docx]

Supplementary Material

**Supplementary Table 2**: Detailed illustration of the protocol undertaken for data collection from Day -1 (day before diet intervention begins) to Day 15 (end of intervention) of the 2-week residential study

| **WEEK 1** |  |  |
| --- | --- | --- |
|  |  |  |
| Day -1  Arrival at the HNU clinic | 4pm - 5.00 pm | Arrival at the HNU clinic   - Return of faecal sample kit and completed food frequency questionnaire - Anthropometry recorded - Non-fasted blood sample collected - Mood questionnaire administered |
|  |  |  |
| Day 1  Start of study: baseline & postprandial measures | 5.30-6.00 am | Bladder voided and body weight recorded   - Return of completed Mood questionnaire - Spot urine sample collected |
|  | 6.30 am | Phlebotomy Room:  Cannulation + baseline blood sample (T=-60) |
|  | 6.45 am | Body temperature & blood pressure recorded  Start of Indirect Calorimetry: at least 30 min of stable measurements for resting metabolic rate [fasted] |
|  | 7.30 am | Indirect Calorimetry stopped (canopy removed) |
|  | 7.40 am | Blood sample collected, fasted (Baseline, T-5min) |
| Concomitant OGTT + Indirect Calorimetry conducted | 7.40-7.45 am | 75g oral glucose drink administered + once consumed (T=0 min) canopy placed for Indirect Calorimetry re-start [postprandial glucose induced thermogenesis, GIT] |
|  | 8.00-9.30 am | Blood sample collected every 15 min (OGTT; T=15min to T=90 min) |
|  | 9.45 am | Final blood sample collected (T=120 min)   - Cannula removed - Indirect calorimetry stopped; canopy removed - Body temperature & blood pressure recorded |
|  | 10.00 am | Breakfast, HNU clinic dining room |
|  | 1.00 pm | Lunch* |
|  | 4.00 pm | Mid-afternoon snack* |
|  | 7.00-8.00 pm | Dinner, HNU clinic dining room |
|  | 10.00 pm | Bedtime |
|  |  |  |
| Day 2 | 6.30-6.45 am | Bladder voided and body weight recorded   - Spot urine sample collected - Fasted blood sample collected |
|  | 7.00-8.00 am | Breakfast, HNU clinic dining room |
|  | 10.00 am | Mid-morning snack* |
|  | 1.00 pm | Lunch* |
|  | 4.00 pm | Mid-afternoon snack* |
|  | 7.00-8.00 pm | Dinner, HNU clinic dining room |
|  | 10.00 pm | Bedtime |
|  |  |  |
| Day 3 | 6.30am-10.00pm | Body weight recorded, full diet control |
| Day 4 | 6.30am-10.00pm | Body weight recorded, full diet control |
| Day 5 | 6.30am-10.00pm | Body weight recorded, full diet control |
| Day 6 | 6.30am-10.00pm | Body weight recorded, full diet control |
|  |  |  |
| Day 7  Mid-way through study | 6.30-6.45 am | Bladder voided and bodyweight recorded   - Start of 24-hr urine sample collection - Fasted blood sample collected - Mood Questionnaire administered |
|  | 7.00-8.00 am | Breakfast, HNU clinic dining room |
|  | 10.00 am | Mid-morning snack* |
|  | 1.00 pm | Lunch* |
|  | 4.00 pm | Mid-afternoon snack* |
|  | 7.00-8.00 pm | Dinner, HNU clinic dining room; followed by Focus Group/Interview session in HNU Lounge |
|  | 10.00 pm | Bedtime |
|  |  |  |
| **WEEK 2** |  |  |
|  |  |  |
| Day 8 |  | Body weight recorded, full diet control |
| Day 9 |  | Body weight recorded, full diet control |
| Day 10 |  | Body weight recorded, full diet control |
| Day 11 |  | Body weight recorded, full diet control |
| Day 12 |  | Body weight recorded, full diet control |
| Day 13 |  | Body weight recorded, full diet control |
|  |  |  |
| Day 14 | 6.30-6.45 am | Bladder voided and body weight recorded   - Spot urine sample collected - Fasted blood sample collected - Faecal sample collection kit provided (end of study sample) - Mood questionnaire provided |
|  | 7.00-8.00 am | Breakfast, HNU clinic dining room |
|  | 10.00 am | Mid-morning snack* |
|  | 1.00 pm | Lunch* |
|  | 4.00 pm | Mid-afternoon snack* |
|  | 7.00-8.00 pm | Dinner, HNU clinic dining room |
|  | 10.00 pm | Bedtime |
|  |  |  |
| Day 15  End of study: baseline & postprandial measures | 5.30-6.00 am | Bladder voided and body weight recorded   - Return of completed Mood questionnaire - Spot urine sample collected - Faecal sample collected |
|  | 6.30 am | Phlebotomy Room:  Cannulation + baseline blood sample (T=-60) |
|  | 6.45 am | Body temperature & blood pressure recorded  Start of Indirect Calorimetry: at least 30 min of stable measurements for resting metabolic rate [fasted] |
|  | 7.30 am | Indirect Calorimetry stopped (canopy removed)  Body temperature & blood pressure recorded |
|  | 7.40 am | Blood sample collected (Baseline, T-5min) |
|  | 7.40-7.45 am | 75g oral glucose drink administered + once consumed (T=0 min) canopy placed for Indirect Calorimetry re-start [postprandial glucose induced thermogenesis, GIT] |
|  | 8.00-9.30 am | Blood sample collected every 15 min (OGTT; T=15min to T=90 min) |
|  | 9.45 am | Final blood sample collected (T=120 min)   - Cannula removed - Indirect calorimetry stopped; canopy removed - Body temperature & blood pressure recorded |
|  | 10.00 am | Breakfast provided |
|  |  | END of STUDY |

* HNU clinic dining room/packed for take away to work; plus 1.5 L Chinese style tea provided daily.
